# Protective Effect of Methoxsalen on Spinal Cord Injury in the Rat Model via Regulation of the PI3K/Akt Pathway

**DOI:** 10.5812/ijpr-171331

**Published:** 2026-08-08

**Authors:** Mao Zhaohu, Zhang Zheng, Shan Qunqun

**Affiliations:** 1 Department of Spinal Surgery, PLA 960th Hospital, 250000, JiNan, China

**Keywords:** Methoxsalen, Spinal Cord Injury, Inflammation, Oxidative Stress, Network Pharmacology

## Abstract

**Background:**

Spinal cord injury (SCI) is a major cause of disability, and the management of secondary injury remains challenging.

**Objectives:**

This study evaluated the protective effects of methoxsalen against SCI and investigated its potential mechanism of action.

**Methods:**

Network pharmacology and molecular docking were performed to identify the molecular targets of methoxsalen for the treatment of SCI. SCI was induced in rats by laminectomy, and methoxsalen (12 and 24 mg/kg, p.o.) was administered for 14 days after SCI induction. Motor function was assessed using the Basso, Beattie, and Bresnahan (BBB) score, and spinal cord inflammation was evaluated by assessing water deposition and inflammatory cytokine levels in SCI rats. Quantitative reverse transcription polymerase chain reaction (qRT-PCR) was performed to assess the mRNA expression of phosphoinositide 3-kinase (PI3K) and protein kinase B (AKT) in SCI rats.

**Results:**

Molecular docking data showed that methoxsalen interacted with AKT, PI3K, and nuclear factor-κB (NF-κB), with binding energies of -9.1, -9.2, and -9.4 kcal/mol, respectively. The BBB score was significantly improved in the methoxsalen-treated group compared with the SCI group. MDA (18.6 ± 0.9 nmol/mg) and ROS (1.30 ± 0.04-fold) levels were significantly reduced, whereas GSH (92.0 ± 2.2 µM/mg) and SOD (54.2 ± 1.3 U/mg) levels were increased in the methoxsalen-treated group compared with the SCI group. Methoxsalen treatment ameliorated inflammatory alterations and cytokine levels in rats with SCI. The mRNA expression of PI3K, AKT, and NF-κB was attenuated in spinal cord tissue from methoxsalen-treated rats with SCI.

**Conclusions:**

Methoxsalen treatment improved motor function in rats with SCI by modulating the PI3K/AKT signaling pathway.

## 1. Background

Spinal cord injury (SCI) is a traumatic condition that causes neuropathic pain, sensorimotor dysfunction, and permanent disability (1). SCI affects patients’ quality of life, and approximately 10 - 80 million patients are affected each year (2). SCI consists of primary and secondary stages. Primary injury occurs due to physical or chemical damage to the spinal cord, including spinal ligament tears and fragmentation (3). Secondary injury develops after the primary injury and involves molecular dysfunction, autophagy, and oxidative stress (4). Autophagy is a cellular process that recycles damaged cellular components. The regulation of autophagy is essential for recovery from SCI. Several pathways regulate autophagy, including the PI3K/AKT/mTOR pathway, which is substantially involved in SCI (5). mTOR protein downregulation regulates several cellular functions, including autophagy, apoptosis, and cellular proliferation, through the PI3K/AKT pathway (6). Moreover, neuronal function and differentiation in the central nervous system are modulated via the PI3K/AKT pathway (7). These findings suggest that the PI3K/AKT pathway is an important target for the management of SCI.

Natural products isolated from medicinal plants have shown promising effects in the management of several disorders. Furanocoumarins are widely found in medicinal plants and possess pharmacological properties, including anti-inflammatory, antioxidant, and antiproliferative activities (8). Methoxsalen is a furanocoumarin isolated from citrus plants such as lemon (*Citrus limon*) and orange (*Citrus aurantium* L.) (9). Methoxsalen is used to treat proliferative epidermal diseases such as eczema, vitiligo, and psoriasis (10). It has been reported to improve cognitive functions, including learning and memory, by regulating acetylcholinesterase (AChE) activity (11). Moreover, methoxsalen has shown beneficial effects in the management of osteoporosis associated with postmenopausal and diabetic conditions by regulating inflammatory pathways (12). It also has potential antiarrhythmic and antitumor activities (13, 14). A study revealed that methoxsalen ameliorates alterations in the PI3K/Akt pathway in neuroblastoma and colon cancer (15).

## 2. Objectives

The objective of this study was to determine the beneficial effects of methoxsalen in rats with SCI. An in silico study was performed to identify the molecular targets of methoxsalen for SCI. Specifically, this study evaluated the effects of methoxsalen on behavioral changes, inflammatory responses, and the PI3K/Akt pathway in SCI rats.

## 3. Methods

### 3.1. Chemicals

Methoxsalen (PHR3040) was procured from MilliporeSigma, Massachusetts, United States. Enzyme-linked immunosorbent assay (ELISA) kits for cytokines, including interleukin (IL)-1β (88 - 7013A-88), IL-10 (BMS629), IL-18 (BMS267 - 2), and tumor necrosis factor-α (TNF-α) (BMS607 - 3), and the GeneJET RNA Purification Kit (K0731) were purchased from Thermo Fisher Scientific Inc., Massachusetts, United States.

### 3.2. In Silico Study

#### 3.2.1. Target Prediction for Methoxsalen Against SCI

PubChem (https://pubchem.ncbi.nlm.nih.gov/) was used to obtain the 2-dimensional SMILES structural information for methoxsalen, which was then used to predict targets using the SwissTargetPrediction database (https://www.swisstargetprediction.ch/). Genes associated with spinal cord injury were identified using the GeneCards database (https://www.genecards.org). Venny 2.1 (https://bioinfogp.cnb.csic.es/tools/venny/) was used to identify overlapping targets between SCI and methoxsalen. These overlapping targets were used to determine protein-protein interactions using the STRING database (https://string-db.org) at a confidence score of ≥ 0.4. Gene Ontology (GO) and Kyoto Encyclopedia of Genes and Genomes (KEGG) enrichment analyses were performed by entering the identified network targets into SRplot (https://www.bioinformatics.com.cn/en) to identify pathways associated with SCI.

#### 3.2.2. Assessment of Methoxsalen Interaction With the PI3K/AKT Pathway

Docking analysis was performed to determine the interaction between methoxsalen and proteins involved in regulation of the PI3K/AKT pathway. Structural information for methoxsalen was retrieved from the PubChem compound database. Docking analysis was conducted using AutoDock Vina. Protein structures were prepared in Discovery Studio Visualizer v20.1.0.19295 (Dassault Systèmes, San Diego, CA, USA, 2020) by removing HETATM records from the PDB files. Polar hydrogen atoms were added in AutoDock Vina, and the ligand-binding grid was selected using AutoGrid4. Binding energy and hydrogen-bond formation with protein amino acid residues were evaluated using AutoDock Vina.

### 3.3. In Vivo Study

#### 3.3.1. Establishment of the SCI Rat Model

Twenty-four healthy male Sprague-Dawley rats aged 10 - 12 weeks and weighing 190 - 210 g were used. All rats were maintained under standard conditions in accordance with the guidelines and protocols for animal studies approved by the Institutional Ethical Committee of PLA 960th Hospital, China (2025070; January 13, 2025).

The laminectomy method was used to induce SCI as described previously (16). All surgical procedures were performed under strict aseptic conditions. Rats were anesthetized using an appropriate anesthetic regimen to induce deep anesthesia. The dorsal thoracic region was shaved and disinfected with a povidone-iodine solution. Animals were placed in the prone position, and an incision was made over the thoracic vertebral region to expose the vertebral column. Laminectomy was performed at the T9 - T10 level without mechanical damage to the spinal cord tissue, while preserving the dura mater. The exposed T9 - T10 region was impacted with a 2.5-mm impactor tip attached to a spring to generate a force of 200 kilodynes (Kdyn), thereby inducing edema that mimicked secondary injury associated with spinal cord trauma. Animals were placed on warming pads during recovery from anesthesia and were allowed free access to food and water. Food intake, body weight, hydration status, and general health were monitored daily throughout the study. All animals survived the surgical procedure.

The 24 rats were divided into 4 groups of 6 animals each. The sample size was selected according to previously reported studies (17), and 6 animals per group were considered sufficient for statistical analysis of the study protocol and molecular end points. A formal power analysis was not performed. The sham group received anesthesia and surgery without laminectomy and received vehicle treatment. The negative control (NC; disease control) group received vehicle treatment. The methoxsalen groups received methoxsalen at 12 or 24 mg/kg p.o. at a volume of 10 mL/kg (11) for 14 days, beginning 24 hours after SCI induction. Methoxsalen was suspended in 5% carboxymethyl cellulose (CMC) and administered orally at 10 mL/kg; the sham and NC groups received 0.5% CMC as the vehicle. Animals with accidental injuries unrelated to the study protocol or severe illness during the study were excluded. To minimize the risk of bias, investigators responsible for assessing study parameters were blinded to group allocation.

#### 3.3.2. Determination of Motor Function

Motor function was assessed in all groups using the Basso, Beattie, and Bresnahan (BBB) method (18). BBB scores were assessed on days 1, 3, 7, and 14. The BBB scale ranges from 0 to 21, with a score of 0 indicating a complete absence of hindlimb function and a score of 21 indicating normal locomotor function, as shown in Table 1. BBB scores were assessed by 2 independent individuals blinded to treatment-group allocation.

**Table 1.** Basso, Beattie, and Bresnahan Locomotor Scale Rating and Description of Each Function

Scores	Description of Locomotor Function
**0**	No movement of the hindlimb
**1**	Slight movement of 1 or 2 joints
**2**	Extensive movement of 1 joint
**3**	Extensive movement of 2 joints
**4**	Slight movement of all 3 joints (hip, knee, ankle)
**5**	Slight movement of 2 joints and extensive movement of 1 joint
**6**	Extensive movement of 2 joints and slight movement of 1 joint
**7**	Extensive movement of all 3 joints
**8**	Sweeping of the hindlimb without weight support
**9**	Plantar placement of the paw without weight support
**10**	Occasional weight-supported plantar steps, no coordination
**11**	Frequent weight-supported plantar steps, no coordination
**12**	Frequent weight-supported plantar steps with occasional forelimb-hindlimb coordination
**13**	Consistent weight-supported plantar steps with coordination
**14**	Consistent coordinated gait with occasional toe clearance
**15**	Consistent coordinated gait with frequent toe clearance
**16**	Consistent coordinated gait with parallel paw placement
**17**	Consistent coordinated gait, predominantly parallel paw placement, trunk instability
**18**	Consistent coordinated gait with trunk stability and tail consistently up
**19**	Normal gait with consistent trunk stability and tail position
**20**	Near-normal locomotion
**21**	Normal locomotor function

#### 3.3.3. Determination of Water Content in the Spinal Cord

The wet/dry method was used to determine spinal cord water content. All rats were sacrificed, and spinal cord tissue was collected and weighed immediately to obtain the wet weight. The tissue was then dried at 95 °C for 3 days and weighed to determine the dry weight. Water content in the spinal tissue was calculated using the following formula:

Water content (%) = Wet weight/Dry weight × 100%.

#### 3.3.4. Determination of Blood-Spinal Cord Barrier Leakage

Evans blue (EB) effusion was used to assess blood-spinal cord barrier leakage. EB was administered through the femoral vein. One hour later, spinal tissue samples were isolated after transcardial perfusion with physiological buffered saline (0.1 M). Spinal cord tissue was homogenized in 3 mL of 50% trichloroacetic acid, and the supernatant was separated after centrifugation at 12,000 rpm for 20 minutes. A 1-mL supernatant sample was treated with trichloroacetic acid solution (1:3) and 1 mL of ethyl alcohol at 4 °C overnight. Samples were analyzed at excitation and emission wavelengths of 620 and 680 nm, respectively, using a microplate reader. A standard curve was used to convert the measured values to EB content.

#### 3.3.5. Preparation of Spinal Tissue Homogenate

Spinal tissue (50 - 100 mg) was isolated from each animal and homogenized in ice-cold PBS/lysis buffer (1:9, w/v) at 4 °C using a tissue homogenizer. The supernatant was separated after centrifugation at 8,000 - 10,000 rpm for 10 - 15 minutes and used for subsequent biochemical analyses.

#### 3.3.6. Determination of Cytokines

Spinal cord tissue homogenate was used to determine the levels of cytokines, including IL-1β, IL-6, IL-12, and TNF-α, by ELISA according to the kit supplier's instructions.

#### 3.3.7. Assessment of Oxidative Stress Parameters

Oxidative stress parameters, including malondialdehyde (MDA), superoxide dismutase (SOD), and glutathione peroxidase (GSH), were estimated in tissue homogenates from SCI rats. The method of Ohkawa et al. was used to estimate MDA levels; biological samples were reacted with thiobarbituric acid, and absorbance was measured at 532 nm (19). The method reported by Misra and Fridovich was used to estimate SOD activity; biological samples were treated with epinephrine, and absorbance was measured at 480 nm to assess changes in SOD activity (20). The method reported by Flohé and Günzler was used to estimate glutathione peroxidase; biological samples were reacted with t-butyl hydroperoxide, and absorbance was measured at 340 nm (21). Reactive oxygen species (ROS) levels were determined using ELISA.

#### 3.3.8. qRT-PCR

Total RNA was extracted from isolated spinal cord tissue from SCI rats using the GeneJET RNA Purification Kit according to the manufacturer's instructions. cDNA was reverse-transcribed from isolated RNA using a cDNA synthesis kit. Gene amplification was performed using SYBR Green Select PCR Master Mix by RT-PCR. Primers were procured using the sequences shown in Table 2. RT-PCR was performed with an activation step of 15 minutes at 95 °C, followed by 10 seconds at 95 °C, 15 seconds at 65 °C, and 30 seconds at 72 °C for 45 cycles. The 2^−ΔΔCT^ method was used to calculate relative gene mRNA expression.

**Table 2.** Primer Details

Sr. No.	Primer	Forward	Reverse
**1**	Akt	GAGATGGATGCGTCTACAACCC	TCCACTTGCCTTCTCTCGAACC
**2**	PI3K	ACACCACGGTTTGGACTATGG	GGCTACAGTAGTGGGCTTGG
**3**	NF-κB	GCTGCCAAAGAAGGACACGACA	GGCAGGCTATTGCTCATCACAG
**4**	GAPDH	GGATACTGAGAGCAAGAGAGA	TTATGGGGTCTGGGATGGAA

#### 3.3.9. Statistical Analysis

Data are presented as mean ± SEM (n = 6) and were analyzed using GraphPad Prism 8.2.1 (GraphPad Software, USA). One-way analysis of variance (ANOVA), followed by Bonferroni post hoc testing, was used for cytokine, oxidative stress, and qRT-PCR data measured at a single time point. Two-way repeated-measures ANOVA followed by Bonferroni post hoc testing was used to analyze BBB scores measured repeatedly on days 1, 3, 7, and 14 after SCI induction. Statistical significance was set at P < 0.05.

## 4. Results

### 4.1. Potential Targets of Methoxsalen Against SCI

Methoxsalen was searched on the NCBI website using CAS number 298 - 81 - 7 (PubChem CID: 4114), with the 2-dimensional SMILES structure COC1=C2C(=CC3=C1OC=C3)C=CC(=O)O2. A total of 100 protein targets for methoxsalen were identified using the SwissTargetPrediction database, as shown in Table 3.

**Table 3.** Protein Targets of Methoxsalen Identified by the SwissTargetPrediction Database

ACHE	PTK2	SRC	TMIGD3	RPS6KA2
**CYP1A2**	PLK1	MKNK1	ALPL	CCR1
**CA12**	CSNK2A1	TLR4	RPS6KB1	PTAFR
**CA9**	PLK4	EIF2AK2	CBR1	HRH3
**KCNA3**	MAP3K8	GSR	IDO1	HRH4
**CA7**	BRAF	CCR4	PTPRG	CCR2
**CA13**	EPHB4	EDNRA	OXTR	IKBKB
**CA2**	HSPA1A	ICAM1	ACE	MAPK14
**MAOA**	NUAK1	SELE	ERBB2	CYP11B1
**EGFR**	SQLE	BACE1	FASN	CYP11B2
**CA1**	FGR	PPARA	F13A1	PTK2B
**NFKB1**	LYN	ADRA2A	TSPO	CYP19A1
**TAAR1**	AURKB	ADRA2C	PARP1	PDE8B
**DAO**	AURKA	ADRA2B	PARP2	XDH
**MAOB**	AOC3	ADRA1B	MCHR1	ILK
**ADORA1**	NTRK1	PPP1CA	PIK3CA	NFKBIA
**ADORA2B**	CCNE2 CDK2 CCNE1	ELANE	MKNK2	RELA
**ADORA3**	PDE7A	GPR35	CLK1	TERT
**JAK1**	PDE10A	FLT4	BRSK2	NR3C2
**JAK2**	EPHX1	CSNK1D	DYRK3	SGK1

**Figure 1.**
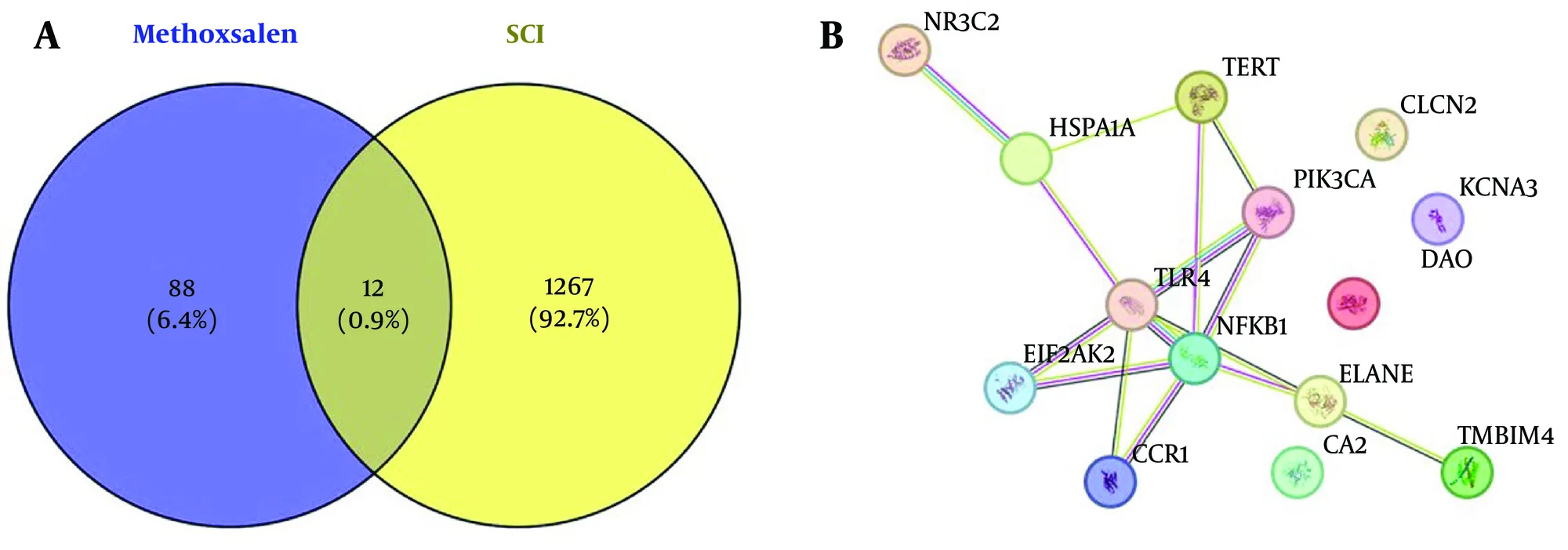
Assessment of molecular targets of methoxsalen against SCI using network pharmacology. A, Assessment of overlapping targets associated with methoxsalen (SwissTargetPrediction database) and SCI (GeneCards database). B, Assessment of the protein-protein interaction (PPI) network of the 12 overlapping SCI targets using the STRING web server at a threshold of 0.4.

A total of 1,279 genes related to spinal cord injury were identified using the GeneCards database. Overlap analysis between these genes and the identified methoxsalen target proteins revealed 12 overlapping proteins (Figure 1A). Protein-protein interactions (PPI) among these overlapping proteins were assessed at a confidence threshold of 0.4 using the STRING database. The PPI network showed 14 edges and 14 nodes, with an average node degree of 2.14, an average local clustering coefficient of 0.424, and a PPI enrichment P value of 0.000461 (Figure 1B).

**Figure 2.**
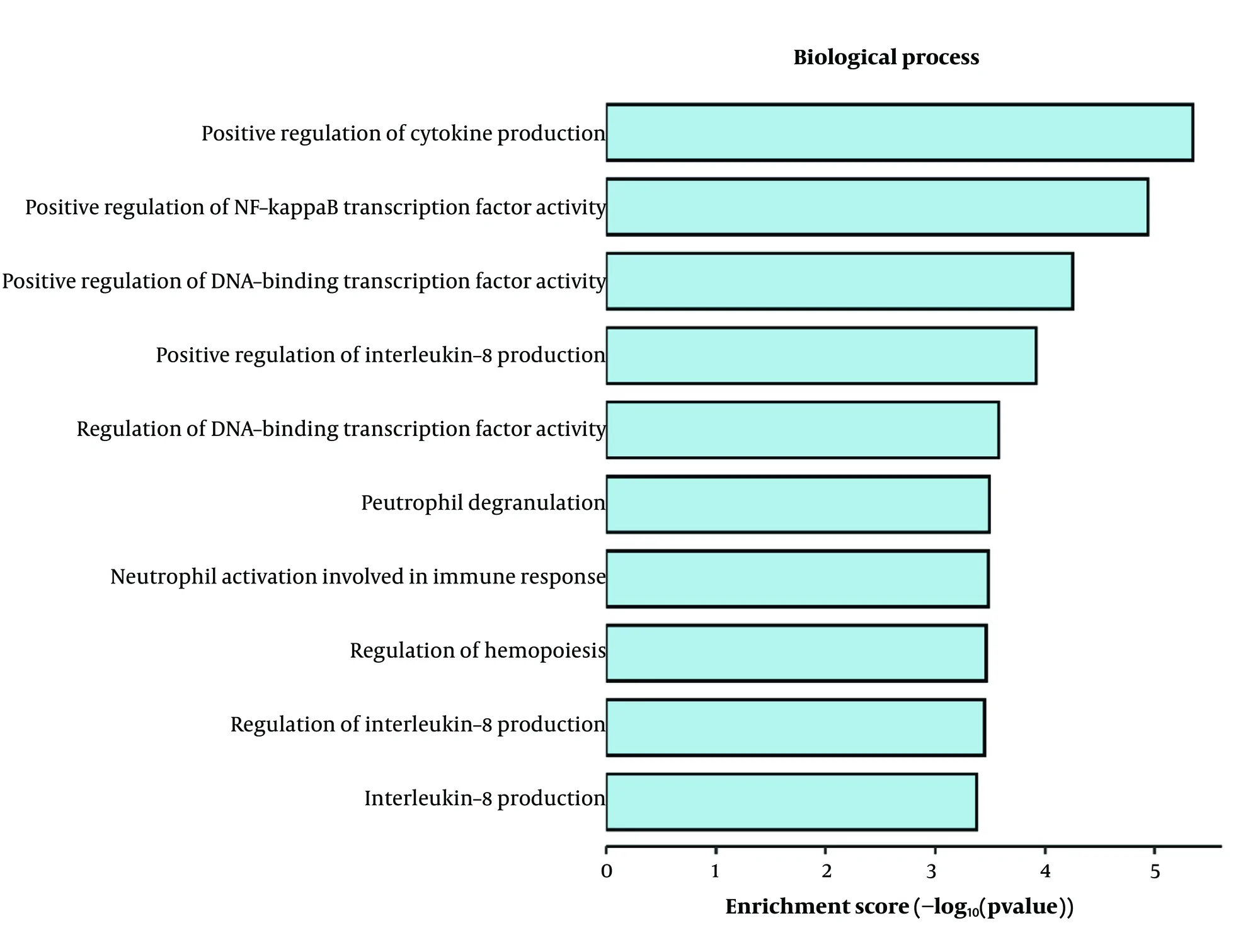
Determination of the possible molecular mechanism of methoxsalen against SCI through gene ontology analysis using SRplot.

GO and KEGG pathway analyses were conducted to identify potential targets of methoxsalen against SCI, as shown in Figure 2. Several targets were identified by GO analysis, and KEGG pathway analysis identified AKT, PI3K, and NF-κB as common targets in the PI3K-AKT pathway in SCI (Figure 1 in the Supplementary File).

### 4.2. Assessment of Docking of Methoxsalen with PI3K, AKT, and NF-κB Proteins

**Figure 3.**
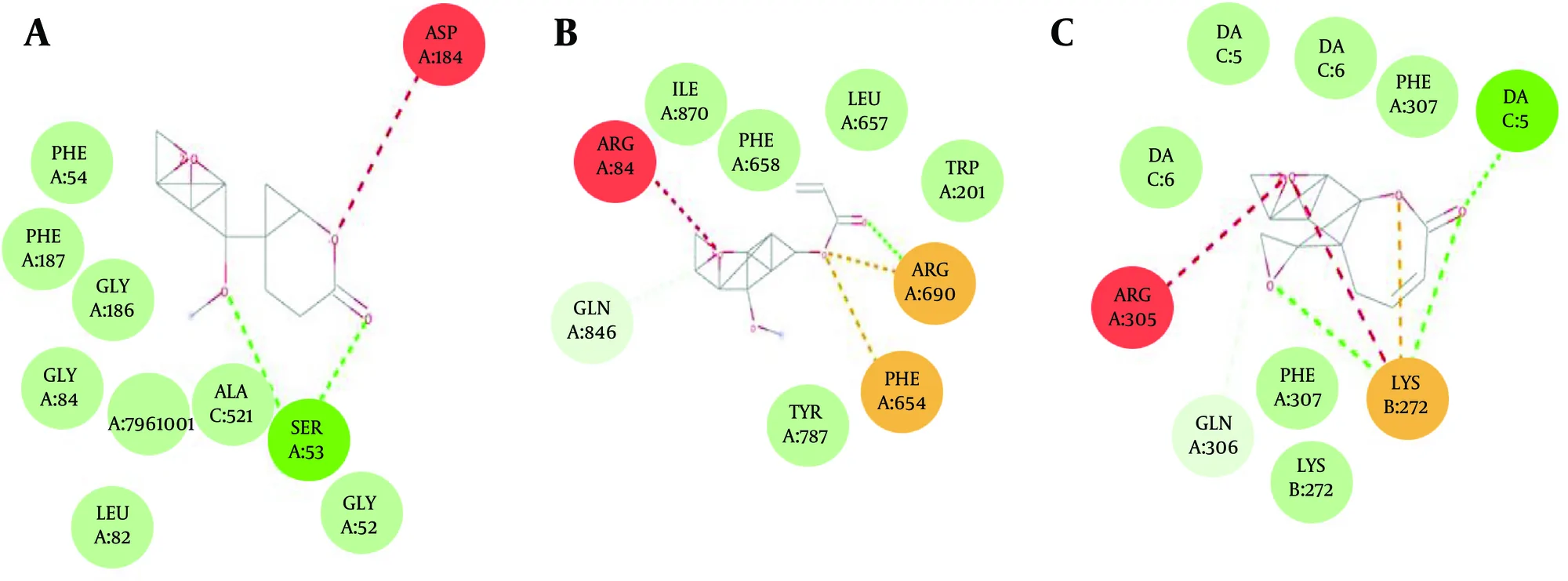
Molecular interactions between methoxsalen and molecular targets, including PI3K, AKT, and NF-κB proteins, assessed by docking with AutoDock Vina. A, Molecular interaction of methoxsalen with AKT; B, molecular interaction of methoxsalen with PI3K; and C, molecular interaction of methoxsalen with NF-κB.

Docking analysis was performed to assess molecular interactions between methoxsalen and PI3K, AKT, and NF-κB proteins, as shown in Figure 3, panels A-C, and Table 4. Methoxsalen interacted with AKT with a binding energy of -9.1 kcal/mol and formed a hydrogen bond with SER A:53. Molecular interactions of methoxsalen with PI3K and NF-κB showed binding energies of -9.2 kcal/mol (ARG A:690) and -9.4 kcal/mol (DA D:5; LYS A:272), respectively.

**Table 4.** Ligand-Protein Interactions Assessed for H-Bond Formation with Protein Residues

Sr. No.	Ligand	Protein	Binding Energy (kcal/mol)	Amino Acid
**1**	Methoxsalen	AKT	-9.1	SER A:53
**1**	Methoxsalen	PI3K	-9.2	ARG A:690
**1**	Methoxsalen	NF-κB	-9.4	DA D:5, LYS A:272

### 4.3. Methoxsalen Attenuates Motor Dysfunction

**Figure 4.**
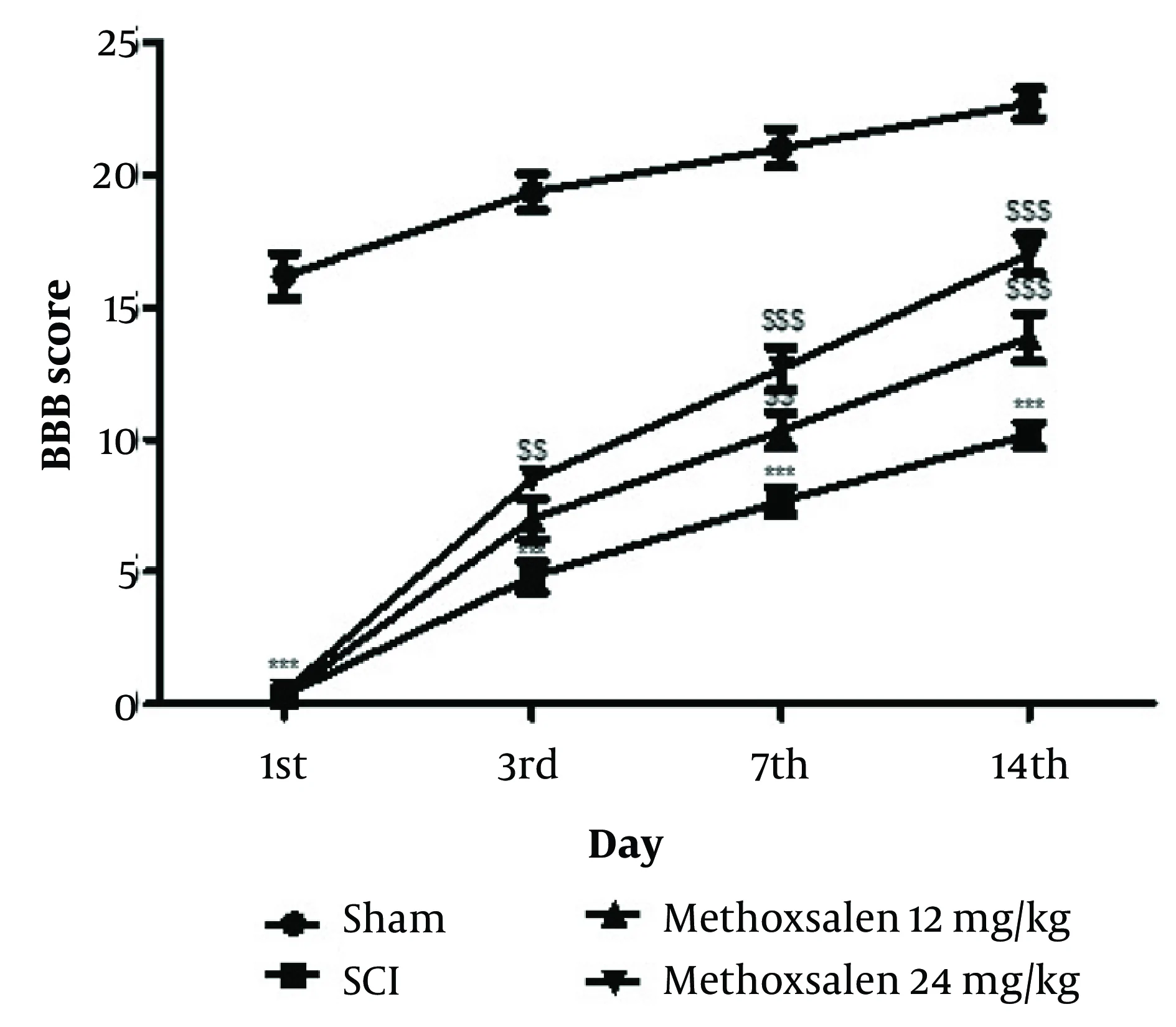
Effect of methoxsalen on motor function in the SCI rat model, assessed using BBB scores on days 1, 3, 7, and 14. Mean ± SEM (n = 6); ***P < 0.001 vs the sham group; $$P < 0.01 and $$$P < 0.001 vs the SCI group.

BBB scores were determined to assess the effect of methoxsalen on motor function in SCI rats on days 1, 3, 7, and 14, as shown in Figure 4. BBB scores were significantly lower in the SCI group than in the sham group on days 1, 3, 7, and 14; this reduction was reversed approximately twofold by methoxsalen treatment in a dose-dependent manner.

### 4.4. Methoxsalen Attenuates Water Content and EB Effusion

**Figure 5.**
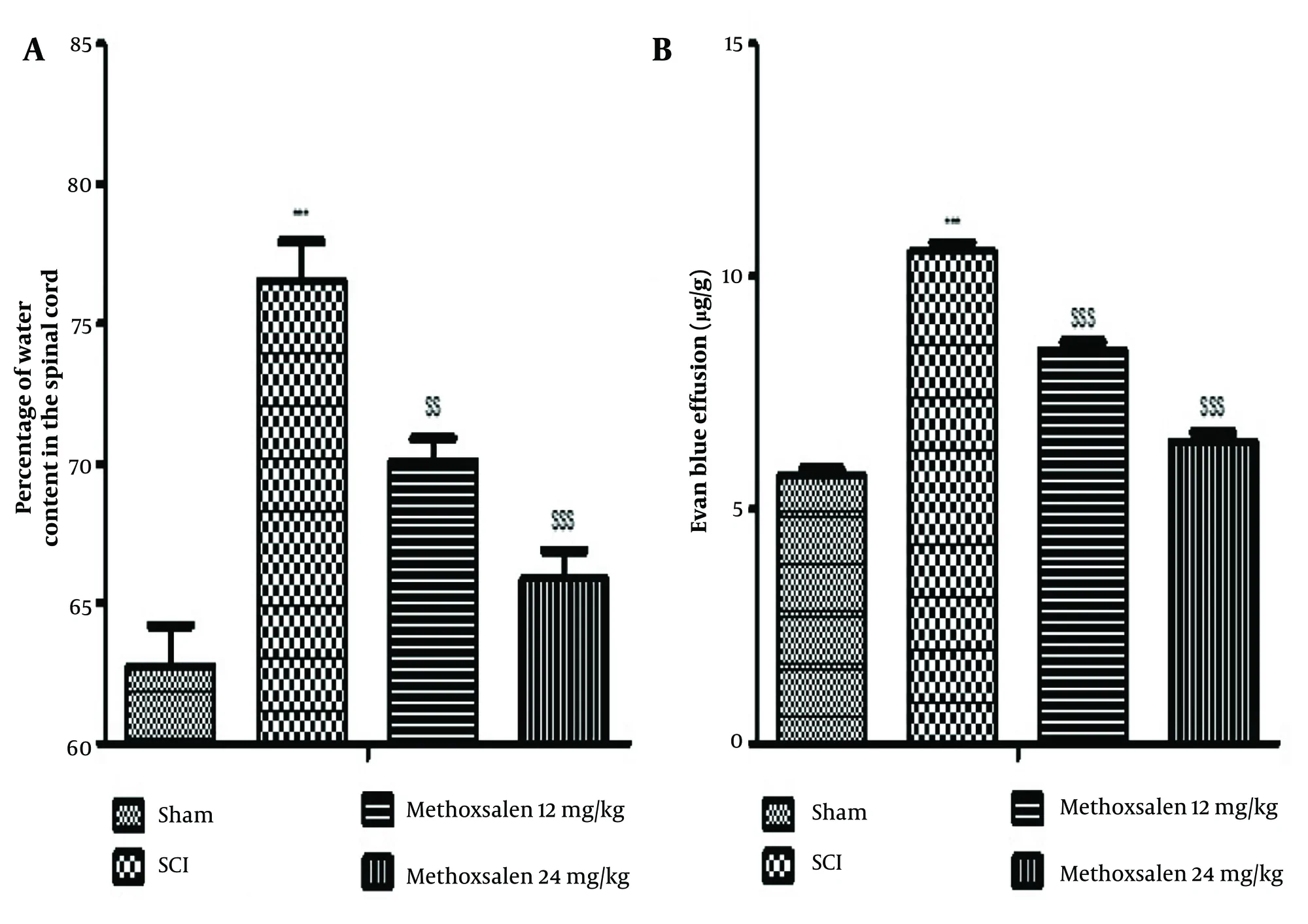
Effect of methoxsalen on spinal cord inflammation in the SCI rat model. A, Percentage water content in the spinal cord of SCI rats; B, EB effusion in the spinal cord of SCI rats. Mean ± SEM (n = 6); ***P < 0.001 vs the sham group; $$P < 0.01 and $$$P < 0.001 vs the SCI group.

The effect of methoxsalen on spinal cord inflammation was assessed by determining the percentage water content and EB effusion in SCI rats (Figure 5A and B). Water content increased significantly to 76.51 ± 1.43%, and EB effusion increased to 10.55 ± 0.18 in the SCI group compared with the sham group (water content, 62.74 ± 1.48%; EB effusion, 5.78 ± 0.14). Methoxsalen treatment significantly reduced water content to 65.87 ± 1.03% and EB effusion to 6.47 ± 0.18 in SCI rats.

### 4.5. Methoxsalen Attenuates Oxidative Stress Parameters

**Figure 6.**
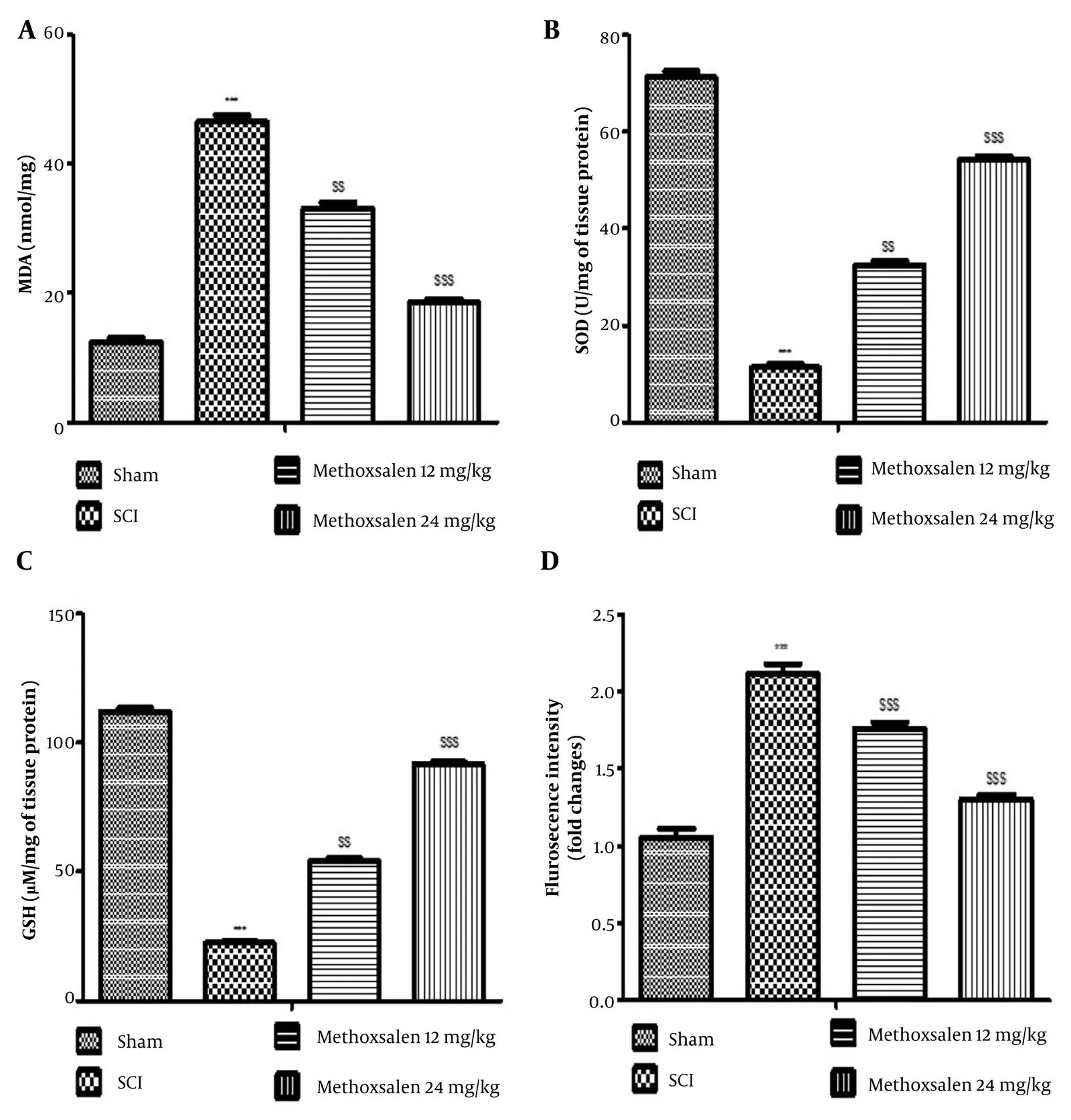
Effect of methoxsalen on oxidative stress parameters in the SCI rat model. A, MDA level; B, SOD activity; C, GSH level; and D, ROS fluorescence intensity. Mean ± SEM (n = 6); ***P < 0.001 vs the sham group; $$P < 0.01 and $$$P < 0.001 vs the SCI group.

Oxidative stress parameters, including MDA, SOD, GSH, and ROS, were estimated in spinal tissue from methoxsalen-treated SCI rats, as shown in Figure 6, panels A-D. MDA (46.58 ± 1.04 nmol/mg) and ROS (2.11 ± 0.06-fold) levels were significantly increased (P < 0.001), whereas GSH levels (22.40 ± 0.55 µM/mg) were reduced in spinal tissue homogenates from the SCI group compared with the sham group (MDA, 18.55 ± 0.57 nmol/mg; ROS, 1.30 ± 0.03-fold; GSH, 112.2 ± 1.42 µM/mg). SOD activity (11.33 ± 0.72 U/mg) was also significantly reduced (P < 0.001) in tissue homogenates from the SCI group compared with the sham group (71.38 ± 1.20 U/mg). Methoxsalen treatment ameliorated these oxidative stress alterations in SCI rats.

### 4.6. Methoxsalen Attenuates Cytokines

**Figure 7.**
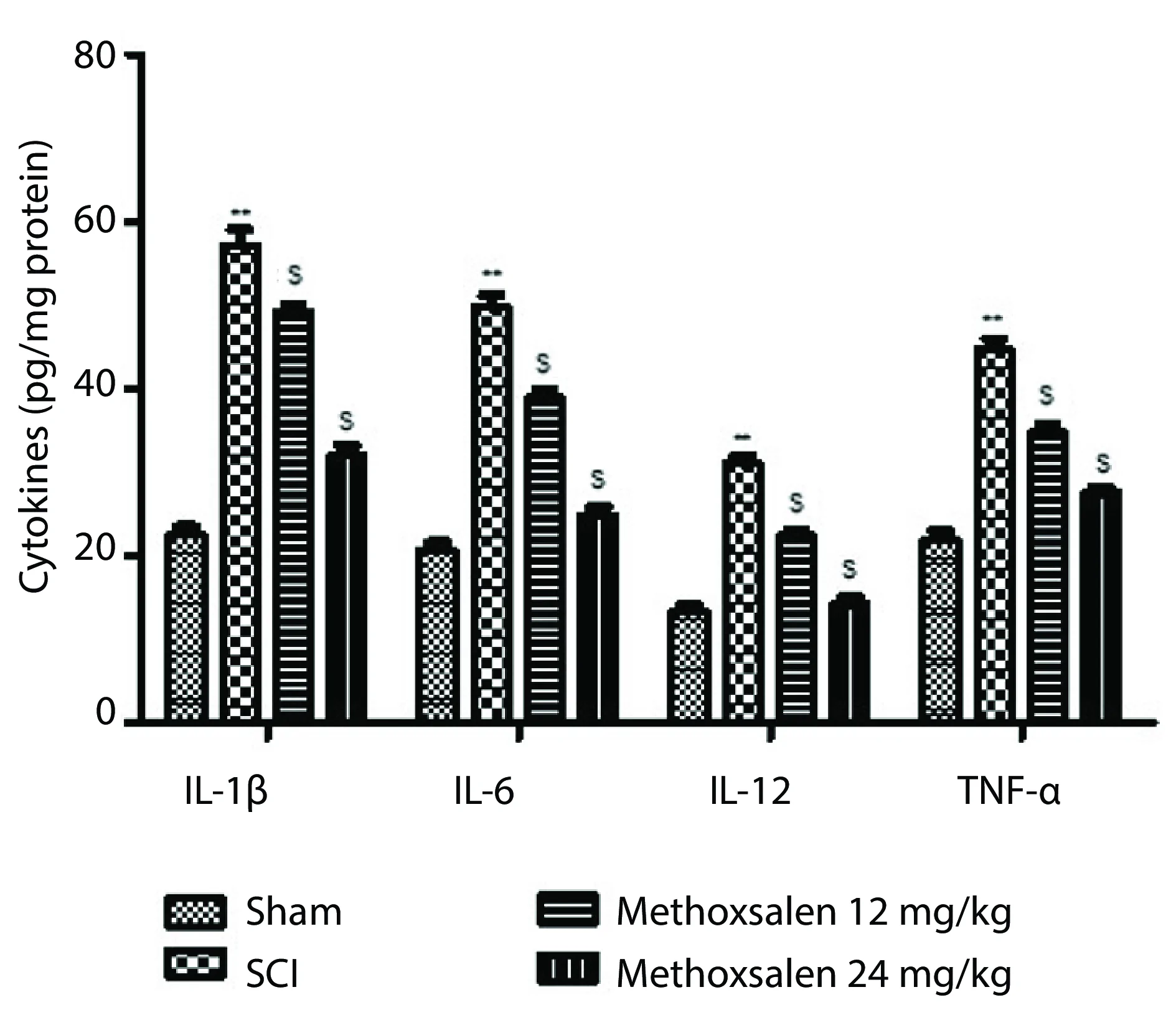
Effect of methoxsalen on IL-1β, IL-6, IL-12, and TNF-α levels in spinal cord tissue homogenates from SCI rats. Mean ± SEM (n = 6); **P < 0.001 vs the sham group; $P < 0.05 and $$P < 0.01 vs the SCI group.

Inflammatory cytokines, including IL-1β, IL-6, IL-12, and TNF-α, were estimated in methoxsalen-treated SCI rats, as shown in Figure 7. Cytokine levels were significantly increased approximately twofold (P < 0.001) in spinal tissue from the SCI group compared with the sham group. Cytokine levels in spinal tissue were significantly reduced approximately onefold in the methoxsalen-treated group compared with the sham group.

### 4.7. Methoxsalen Attenuates mRNA Expression of PI3K, AKT, and NF-κB

**Figure 8.**
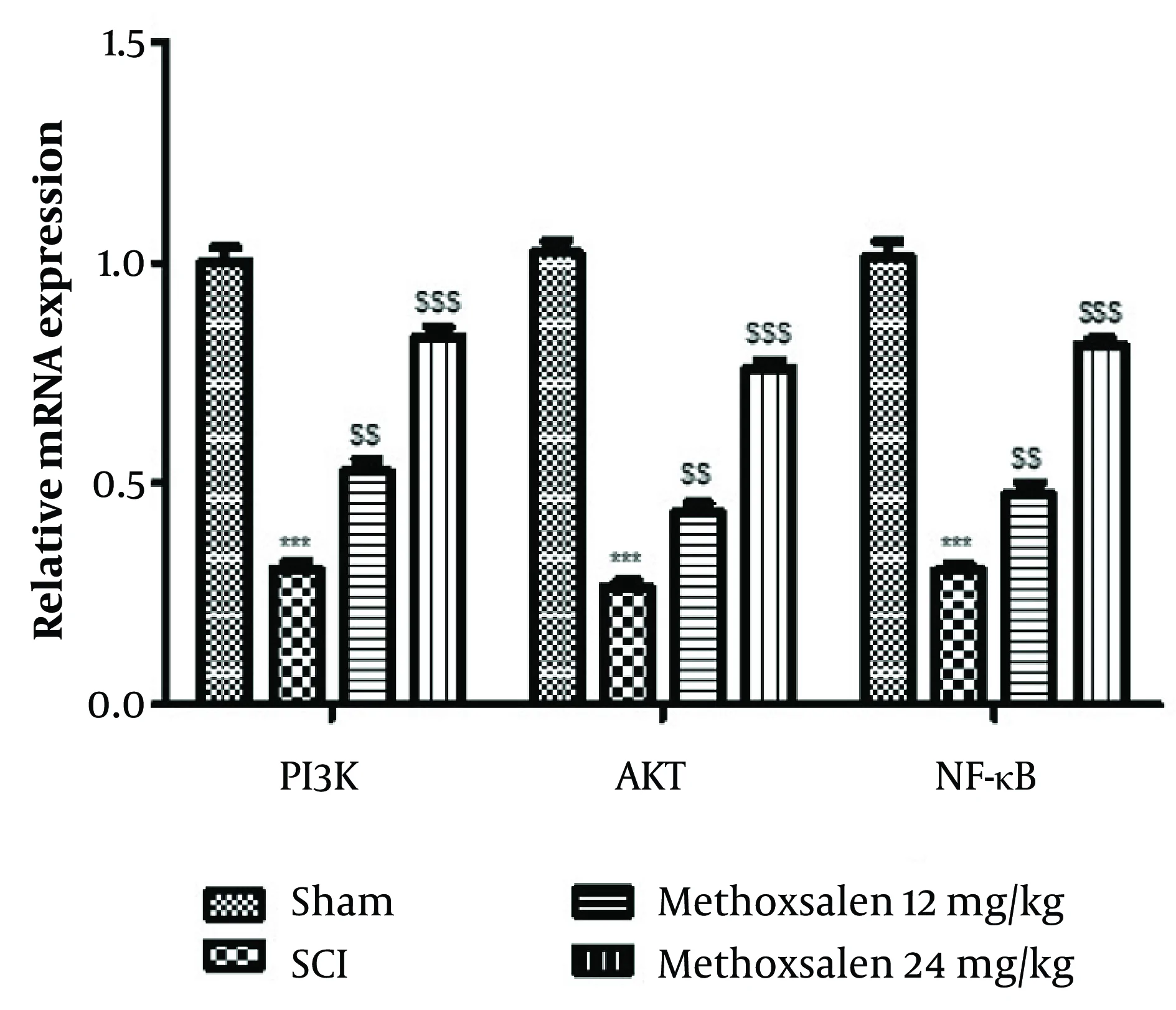
Effect of methoxsalen on mRNA expression of PI3K, AKT, and NF-κB in spinal cord tissue homogenates from SCI rats. Mean ± SEM (n = 6); ***P < 0.001 vs the sham group; $$P < 0.01 and $$$P < 0.001 vs the SCI group.

mRNA expression of PI3K, AKT, and NF-κB was estimated in spinal tissue from methoxsalen-treated SCI rats. There was a significant fourfold reduction in mRNA expression of PI3K, AKT, and NF-κB in spinal tissue from the SCI group compared with the sham group, which was reversed to threefold in methoxsalen-treated SCI rats (Figure 8).

## 5. Discussion

Spinal cord injury is a major cause of physical disability. Mechanical and physical damage to the spinal cord can result in loss of locomotion, stimulus perception, sensation, and sphincter control (22), leading to poor quality of life and imposing an economic burden on affected individuals. These characteristics underscore the need for new therapies for SCI. Therefore, this study evaluated the beneficial effects of methoxsalen against SCI in a rat model.

Physical injury to the spinal cord damages nerves and impairs motor function. Laminectomy-induced SCI in rat models exhibits features that resemble the clinical features of SCI (17). The BBB score effectively assesses motor function and is reduced in animals with SCI (23). The literature suggests that effective SCI management is characterized by improved motor function because prevention of nerve damage promotes normal muscular function (24). In the present study, BBB scores were significantly reduced in the SCI group compared with the sham group and were reversed by methoxsalen treatment in SCI rats.

Inflammation is triggered after physical injury and promotes fluid accumulation at the injury site, resulting in edema. The inflammatory process disrupts endothelial cell membranes and increases fluid deposition in tissue (25). Epithelial cell membrane permeability and water accumulation increase in injured tissue (26), and the present findings support this observation. Control of inflammation is associated with reduced water content through decreased tissue permeability, and methoxsalen treatment reduced these parameters in SCI rats. Tissue injury promotes the synthesis of inflammatory cytokines such as IL-1β, IL-6, IL-12, and TNF-α, which are also involved in secondary injury associated with SCI (27). These cytokines contribute to vasodilation, which increases tissue permeability and contributes to further injury. Drugs used for SCI management reduce cytokine levels in spinal tissue, and methoxsalen treatment attenuated the increased cytokine levels in spinal tissue of SCI rats.

Secondary injury in SCI begins with the production of reactive oxygen species, which generate superoxide anions (28). These superoxide anions damage cell membranes and increase malondialdehyde levels; similarly, the present study showed increased MDA levels in the SCI group compared with the sham group. Superoxide anions are metabolized by superoxide dismutase, and oxidative stress develops when SOD activity is reduced; SOD activity is also reduced in SCI (29). Glutathione levels are also reduced under oxidative stress conditions, including SCI. These oxidative stress parameters were altered in SCI rats, and methoxsalen treatment reversed these changes.

Secondary injury associated with SCI occurs because of dysregulation of the cell cycle, oxidative stress, gliosis, and microglial activation (30). Drugs targeting these interconnected pathways protect against neuronal injury and maintain neuronal integrity. Axonal regeneration is restricted and glial scars develop with activation of gliosis. Methoxsalen has been reported to inhibit DNA synthesis in psoriasis, which may prevent cell-cycle progression and thereby prevent excessive astrocyte proliferation. Hypertrophy of glial cells has been reported in injured cells because of abnormal stimulation of cellular proteins. Moreover, microglial stimulation promotes secondary injury by increasing oxidative stress and inflammatory cytokine levels (31). Methoxsalen treatment attenuated altered oxidative stress markers and cytokine levels in SCI rat tissue. This suggests that methoxsalen may modulate microglial activation through inhibition of DNA replication. Collectively, these findings suggest that methoxsalen could be used for the treatment of secondary injury associated with SCI.

Cytokines and oxidative stress in SCI stimulate AKT release to regulate signaling cascades involved in neuronal proliferation, development, growth, and plasticity (32). The PI3K/AKT pathway is also involved in the regulation of nociceptive signals in both the peripheral and central nervous systems. It is activated in damaged peripheral neurons and is involved in the propagation and generation of neuropathic pain in SCI rats (33). Molecular targets of methoxsalen and SCI, including PI3K, AKT, and NF-κB, were identified by network pharmacology and molecular docking analyses. qRT-PCR analysis supported that methoxsalen treatment attenuated altered mRNA expression of PI3K, AKT, and NF-κB in SCI rats. However, this investigation was limited to computational prediction and gene expression changes. The present study showed a protective effect of methoxsalen against SCI through regulation of the PI3K/Akt pathway. The study was restricted to docking analysis and mRNA expression analysis for mechanistic evaluation, which is a major limitation. Protein expression was not evaluated and, therefore, activation or inhibition of the PI3K/Akt pathway could not be confirmed. Future studies should include western blotting and assessment of the phosphorylation status of these molecular proteins by immunofluorescence to validate the proposed mechanism.

### 5.1. Conclusions

Methoxsalen improved locomotor function by ameliorating inflammatory responses and oxidative stress in SCI rats. Network pharmacology identified potential molecular targets of methoxsalen against SCI, including PI3K, Akt, and NF-κB, and methoxsalen interacted effectively with these targets. The in vivo findings showed that neuronal function and nociceptive responses improved significantly with methoxsalen treatment in SCI rats, as it ameliorated the altered PI3K/AKT signaling pathway.

ijpr-25-1-171331-s001.pdf

## Data Availability

The dataset presented in the study is available on request from the corresponding author during submission or after publication.
