# Supplementary material for: Protective Effect of Methoxsalen on Spinal Cord Injury in the Rat Model via Regulation of the PI3K/Akt Pathway
Source: Iran J Pharm Res. 2026 Aug 8;25(1):e171331. doi: 10.5812/ijpr-171331 (PMC13589461; doi:10.5812/ijpr-171331)
Supplement: Supplementary file 1 [file ijpr-25-1-171331-s001.pdf]

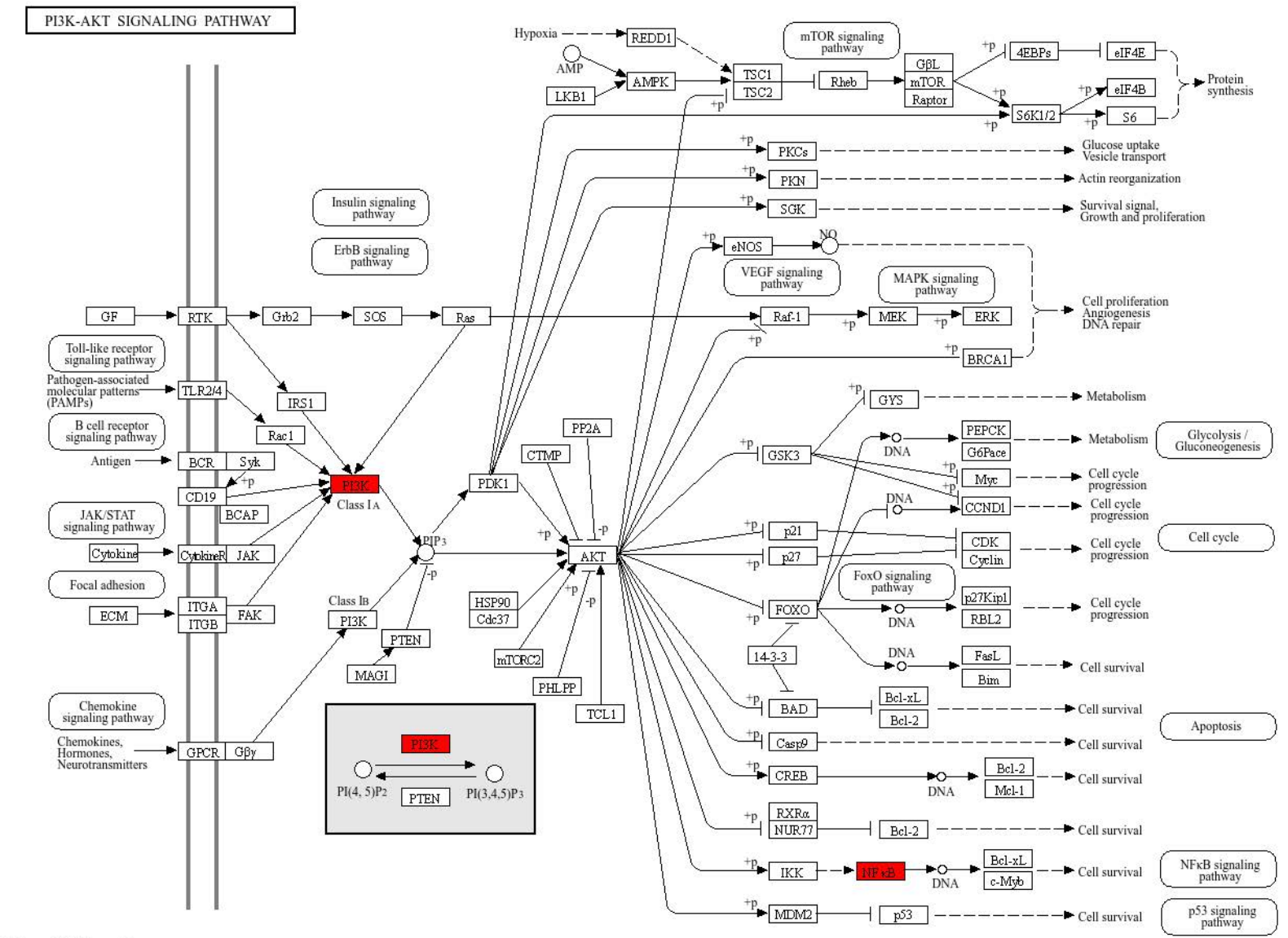

Data on KEGG graph
Rendered by Pathview
